# Some Reflex Disorders of Dental Origin

**Published:** 1898-11

**Authors:** Henry H. Burchard

**Affiliations:** Philadelphia


### International Dental Journal.

Vol. XIX. November, 1898. No. 11.

### Original Communications.[1]

### SOME REFLEX DISORDERS OF DENTAL ORIGIN.[2]

BY HENRY H. BURCHARD, M.D., D.D.S., PHILADELPHIA.

Reflex disorders of dental origin cover a wide field in both sensory and motor disturbances throughout the body, varying in latitude from pain referred from one tooth to a neighboring tooth, to referred pain in a limb terminal, from slight twitching of near muscles, to pronounced epileptiform seizures or paralysis. It is impossible in the limits of a small essay to consider all of the manifestations of nervous disturbance having their origin in dental diseases; nor, indeed, is it advisable to do so, as many of the cases are but rare clinical curiosities. There are, however, features of some degree of constancy which have occupied the attention of nearly every dental practitioner. These are the reflex disturbances arising from diseases of the pulp. Many of the cases of reflex disorders recorded in literature are difficult of classification, because reported by the medical practitioner, who, so far as I have been able to determine, has never differentiated diseases of the pulp from those of the pericementum. Reflex pains do occur in connection

[1] The editor and publishers are not responsible for the views of authors of papers published in this department, nor for any claim to novelty, or otherwise, that may be made by them. No papers will be received for this department that have appeared in any other journal published in the country.

[2] Read before the American Academy of Dental Science, February 2, 1898.

with diseases of the pericementum, but by far the greatest number of cases are found associated with diseases of the pulp. The reason for this is fully and clearly set forth by Dr. Black in Volume I. of "The American System of Dentistry."

The pulp in its normal condition does not possess the tactile sense, and, like similar organs, refers irritation to which it is subjected to some other point.

Such organs, however, do exhibit some degree of constancy as to the point of reference. For example, as pointed out by Black, affections of the iris have an almost constant point of reference to the brow, those of the hip-joint to the inside of the knee, and so on.

This same constancy is observed in connection with the vast majority of acute pulp-diseases; the pain, being rarely localized in the affected tooth (except in cases of acute and sthenic pulpitis), is referred to some point or points of the corresponding nerve-trunk of the same side, the general rule being that the affections of the pulps of the lower teeth have pain reflected to some point of the course of the inferior maxillary nerve, frequently in the auriculo-temporal branch. Pulp-diseases of the upper teeth are most commonly attended by pain in some point of the superior maxillary nerve.

In connection with either, pain may be referred to the first branch of the fifth nerve; this latter reflection is to be regarded as the first of the remote references.

The next reference is to the ear, where not only sensory disturbances may be noted, but also those of special sense. Next in point of frequency are affections of the eye. While many of these are clearly traceable to reflex disturbance of the ciliary ganglion, there are others in which the second cranial nerve is involved. That is to say, while pain is usually confined to the ramifications of the fifth nerve, it may be referred to other nerves whose function is then disturbed; to the second, resulting in functional diseases of the retina; to the third, fourth, sixth, and seventh, in which cases motor disturbances of the muscles about the eye and head are noted; to the eighth, producing disorders of hearing; to the ninth and tenth, when disorders of the tongue, pharynx, larynx, and other parts may be noted, as recorded in dental literature.[1]

The spinal nerves may be affected, resulting in painful diseases of distant parts,—the uterus, a thumb, or even a toe. In addition

---

[1] Brubaker, American System of Dentistry, vol. iii.; Lauder Brunton, Proceedings of the Odontological Society of Great Britain, 1880.

to these should be mentioned the functional disorders of the cerebrum itself, cases of which have been recorded.

To generalize: The acute affections of the pulp are those in which wide reflex disorders are rare. In the chronic diseases, notably in connection with chronic degenerative changes, reflex disorders are common; and *vice versa* in diagnosis; localized pains about the jaws point to the more acute diseases of the pulp; distant pains arouse suspicion of chronic diseases of the organ.

The dental diseases to which such pains are traceable may, for convenience, be grouped under four heads,—1. Reflex disorders due to the irritation of the hypersensitivity of dentine. 2. Those associated with acute pulp-diseases. 3. Those arising during the progress of chronic pulp-diseases. 4. Reflex disorders due to diseases of the pericementum.

While it is certain that almost any of these dental causes may be associated with almost any extent of nervous transferrence, it is extremely probable that pains may be and often are ascribed to a dental source where their true origin lies in other organs. The teeth may be the seat as well as the source of reflex pains. This is notably true in patients who suffer from chronic malarial poisoning, from gout, and in some cases from secondary syphilis. In the gout cases, however, it is more than probable that pathological conditions exist in and about the teeth to which general dental pains are due.

The test of a dental origin of a distant pain should be, Does the pain disappear promptly upon the correction of the dental disease without any other treatment? The vast majority of localized pains about the head and face are due to diseases of the eyes and teeth. Those of the eyes are most frequently located in the first division of the fifth nerve, and, as stated, those of the teeth in the second and third branches. These rules are open to many exceptions, but serve as directing signs in the location of the causes of facial and cranial pains.

### REFLEX DISORDERS DUE TO EXPOSED DENTINE.

As a general proposition, it may be stated that reflex disorders due to the irritation of hypersensitive dentine are most frequently associated with its peripheral exposure; furthermore, the most common situations of the points of irritation are upon the necks of the teeth, where a limited amount of cementum has become exposed and removed, laying bare the dentine. This rule is also open to wide exceptions, but is a useful guide. Other situations which should be mentioned are upon abraded surfaces of teeth and in superficial cavities. As in all dental disorders, the extent of the reflexes is governed by peculiarities of the individual, being most pronounced and remote in individuals who present a neuralgic dyscrasia. In the ordinary individual, such a condition as a neck-exposure of dentine may give rise to indefinitely locating pains about the lips or jaws, in the neuralgic dentine, exposure at the neck of one tooth may be the cause of severe trigeminal neuralgia, with painful spots at the points of nerve emergence, the supra-orbital, infraorbital, and mental foramina; severe pain in the eye or in the ear. Cases are recorded when typical neuralgic sore areas have existed and resisted general medication, and been found due to exposed dentine at the neck of a tooth. This exposure may be upon any tooth; more than once it has been located upon the disto-cervical portion of a third molar.

In some of these cases deliberate irritation of the exposed dentine may cause a reflex paroxysm, but, unfortunately for purposes of immediate diagnosis, it more frequently happens that the pain induced is local. However, a casual relationship is made clear when it is noted that the reflex pain disappears soon after a thorough cauterization of the exposed dentine.

A diagnostic sign of the condition is the recurrence of pain upon taking faintly acid or intensely sweet substances into the mouth.

### ACUTE DISEASES OF THE PULP.

While it is true that severe and wide reflex pains may be caused by acute hyperæmia and attacks of acute inflammation of the dental pulp, it is rare in these affections that symptoms directly referred to the maxillary region are absent.

In the first-named disorder the taking of cold water in the mouth is almost immediately followed by a paroxysm of pain, usually definitely located in the region of the posterior, middle, or anterior dental nerves. In acute pulpitis the pains are also directly referred to the maxillary region, although a defined sore spot may present as a reflex at some portion of the face or scalp, in which event the existence of a dental disease may not be even suspected.

It is in connection with repeated venous congestion of the pulp, its chronic inflammation, suppurative and non-suppurative, and still more frequently with the formation of defined calcific masses in the pulp-tissues, that wide reflex disorders are most frequently found.

All of these conditions present one common feature which differentiates them in symptomatology from the acute affections of the pulp,—*i.e.*, a lessening instead of an exaltation of the special temperature sense of the pulp. Black has remarked this in his contributions to the "American System of Dentistry," and it will be found almost constant. The reaction develops a special peculiarity, notably in the later stages of some of these degenerations, and that is a lessening of response to cold application and an increasing response to heat.

The nature and time of response to heat affords a valuable diagnostic sign as to the pathological condition of the pulp. It is most marked in abscess of the pulp, less so in venous hyperæmia, and much delayed in extensive calcifications in the pulp substance. These distinctions may be carried to still further differentiations, but are beyond the scope of the present paper.

The vast majority of the common neuralgias, with defined painful areas about the head, are due to one of these dental disorders. Pain in front of the ear, in the ear, pain over or in the eye, with a disposition to press upon the eyeball, tender spots in the occipital region, or in most of the cases of pain at the points of nerve-emergence, all point to a reflex disorder having a dental origin.

In nearly all of these cases pain is unilateral. Should it be found upon both sides of the head, it leads to the suspicion of teeth on the other side being affected or to the existence of some optical defect, notably uncorrected astigmatism, hyperopia, or myopia.

Much confusion may arise through the existence of both dental disorders and optical defects, so that the general injunction of the ophthalmologist to patients, "Have your teeth examined," is reciprocal. The dentist should advise in neuralgic cases, "Have your eyes examined," but should correct or remove all dental disorders to which reflex neuralgias are attributable.

Reflex cranial and other neuralgias are also associated with diseases of the pericementum; the classes of pericemental affections giving rise to them are both septic and non-septic. As with pulp-diseases, reflex neuralgias are most commonly due to chronic rather than acute pericemental diseases. Of the non-septic cases, a hypertrophy of cementum has been found a frequent cause of some of the most remote cases of reflex neuralgia recorded in medical and dental literature.

As with nodular deposits in the pulp, these growths exist frequently, and give rise to no symptoms whatever; presumably, the reason for reflex irritation when it appears is the pressure of the hypertrophic growth upon nerve fibres. Many of these cases are not diagnosed except by a long and tedious process of exclusion, there being frequently no local symptoms which would point to the existence of the dental condition. At the present day, suspected cases would, of course, be unveiled through a radiograph.

Many reflex neuralgias are directly traceable to the existence of septic conditions about the roots of teeth, the proof of the casual association being brought to light by the disappearance of the reflex disturbance when the septic condition about the root is remedied or the tooth is extracted. Indeed, it is extremely probable that a large class of ill-defined disorders might be traced to this source, the lack of continuity and clearness of histories, dental and otherwise, obscuring the connection between dental and other diseases.

This subject might be expanded into a large monograph, even more extensive than Brubaker's admirable contribution to the "American System of Dentistry." Enough, however, has been said to emphasize the direct and indirect importance of dental health and to furnish material for an evening's discussion.

